# Stent-Induced Inflammation: A Comparative Cross-Sectional Study of Post-Implantation Syndrome in Venous and Arterial Procedures

**DOI:** 10.3390/jcm13195937

**Published:** 2024-10-05

**Authors:** Nur Dikmen, Evren Ozcinar, Ali Ihsan Hasde, Ahmet Kayan, Nadir Polat, Ali Ardakani, Ezel Kadiroğlu Yuruyen, Zeynep Eyileten

**Affiliations:** 1 Cardiovascular Surgery Department, Faculty of Medicine, Ankara University, 06100 Ankara, Turkey; nurdikmen@yahoo.com (N.D.); ahasde@gmail.com (A.I.H.); nadirpolat06@hotmail.com (N.P.); ardakani.ali235@gmail.com (A.A.); elifezelkadiroglu@gmail.com (E.K.Y.); zeyileten@gmail.com (Z.E.); 2 Cardiovascular Surgery Department, Kirikkale High Specialization Hospital, 71300 Kirikkale, Turkey; dr.ahmet.kayan@gmail.com

**Keywords:** Post-implantation syndrome (PIS), venous stenting, arterial stenting, endovascular procedures, inflammatory response

## Abstract

**Background:** Postimplantation syndrome (PIS) is a known inflammatory response following endovascular stent placement, yet comparative data between venous and arterial stenting remains limited. This study seeks to evaluate the incidence, characteristics, and clinical implications of PIS across these two distinct vascular territories. **Methods:** We retrospectively analyzed 191 patients who underwent either venous (n = 36) or arterial (n = 155) stent placement. Data collection encompassed demographic profiles, perioperative laboratory findings, and clinical outcomes. The primary endpoint was the incidence of PIS, defined as the presence of fever (≥38 °C), leukocytosis, and elevated C-reactive protein (CRP) within 30 days postprocedure. Secondary outcomes included length of hospital and ICU stay, incidence of endoleaks, reintervention rates, and 30-day mortality. Comparative statistical analyses were conducted to assess differences between the venous and arterial stent groups. **Results:** PIS was observed more frequently in arterial stent patients, as evidenced by significantly elevated postoperative white blood cell counts at 24 and 48 h (*p* = 0.046 and *p* = 0.014, respectively), along with borderline CRP increases (*p* = 0.052). Fever occurrence peaked at 72 and 96 h postprocedure, predominantly in the arterial cohort. Furthermore, patients with arterial stents had significantly longer hospital stays (5.59 ± 0.46 days vs. 3.42 ± 0.36 days; *p* = 0.0018) and a higher rate of 30-day endoleaks (7.1% vs. 0%; *p* = 0.005). Despite similar mortality and major adverse cardiac event (MACE) rates between groups, arterial stent patients exhibited a greater need for reintervention. While PIS was less common among venous stent recipients, its potential impact on postoperative recovery warrants careful monitoring. **Conclusions:** Arterial stenting is associated with a higher incidence of PIS and a more pronounced systemic inflammatory response, contributing to longer hospitalization and increased postoperative complications. Although venous stent patients experience PIS less frequently, its occurrence should not be overlooked, as it may influence overall recovery and clinical outcomes. Recognition and management of PIS in both venous and arterial stent patients are critical to improving patient care and optimizing procedural success.

## 1. Introduction

Post-implantation syndrome (PIS) is a significant clinical phenomenon that occurs following the placement of vascular stents, marked by a systemic inflammatory response. This syndrome, though widely recognized in arterial stenting, remains less understood in the context of venous stents. The lack of data on venous stents creates a critical gap in understanding how different vascular environments—arterial and venous—affect the development and severity of PIS. Moreover, while arterial stents have long been a cornerstone in managing aorto-occlusive diseases, the inflammatory responses that accompany their implantation, such as fever, elevated white blood cell (WBC) counts, and increased C-reactive protein (CRP) levels, remain a clinical challenge [1,2,3,4,5].

PIS was first characterized by Velazquez in 1990 as a condition involving postoperative fever, elevated WBC counts, and elevated CRP levels without an underlying infection. The syndrome is especially noted following endovascular aneurysm repair (EVAR), and its incidence ranges from 13% to 60% in different studies. Although PIS has traditionally been viewed as a self-limiting condition, emerging research suggests that it can be associated with serious complications, such as prolonged hospital stay, acute kidney injury, and adverse cardiovascular events. This underscores the need for better management strategies, especially in high-risk patients. In the setting of endovascular procedures, such as thoracic endovascular aortic repair (TEVAR) and EVAR, PIS often manifests within the first 72 h post-implantation. The inflammatory response following these procedures can vary significantly depending on factors such as the extent of vascular trauma, type of endograft, operative time, and the volume of contrast medium used. Research has highlighted that complex procedures, like TEVAR for Type B aortic dissection (TBAD), present a higher risk for PIS due to the extensive tissue manipulation and inflammatory cascade triggered by multiple endograft deployments. However, the clinical impact of PIS remains a topic of debate. Studies, including those by Xie et al., have shown mixed results regarding the long-term outcomes of patients with PIS. For instance, while some research suggests that PIS does not significantly affect long-term survival, others have identified PIS as an independent risk factor for major adverse events (MAEs) and all-cause mortality within the first year post-EVAR [1,2].

This study aims to systematically compare the incidence and severity of PIS between venous and arterial stent placements. By analyzing inflammatory profiles and clinical outcomes, we seek to identify the differences in post-implantation inflammatory responses across these vascular environments. Understanding these distinctions could lead to the development of targeted strategies to mitigate PIS, ultimately improving patient outcomes following both venous and arterial stent placements.

## 2. Materials and Methods

### 2.1. Study Design and Patient Selection

This study was designed as a retrospective analysis comparing the incidence and severity of post-implantation syndrome (PIS) following venous and arterial stenting procedures. A total of 191 patients who underwent endovascular stent placement between January 2018 and January 2024 were included. The median follow-up duration was 18 months, with a minimum follow-up of 8 months and a maximum follow-up of 32 months.

The study was conducted in accordance with the guidelines of the Declaration of Helsinki and was approved by the Research Ethics Board of Ankara University (approval date: 19 September 2024, protocol no:2024/577). Written informed consent was obtained from all patients. Demographic and clinical information were extracted from the electronic health record system.

Of these, 36 patients received venous stents and 155 patients underwent arterial stenting. The primary aim was to compare the inflammatory response and clinical outcomes associated with PIS in both groups.

**Figure 1:**
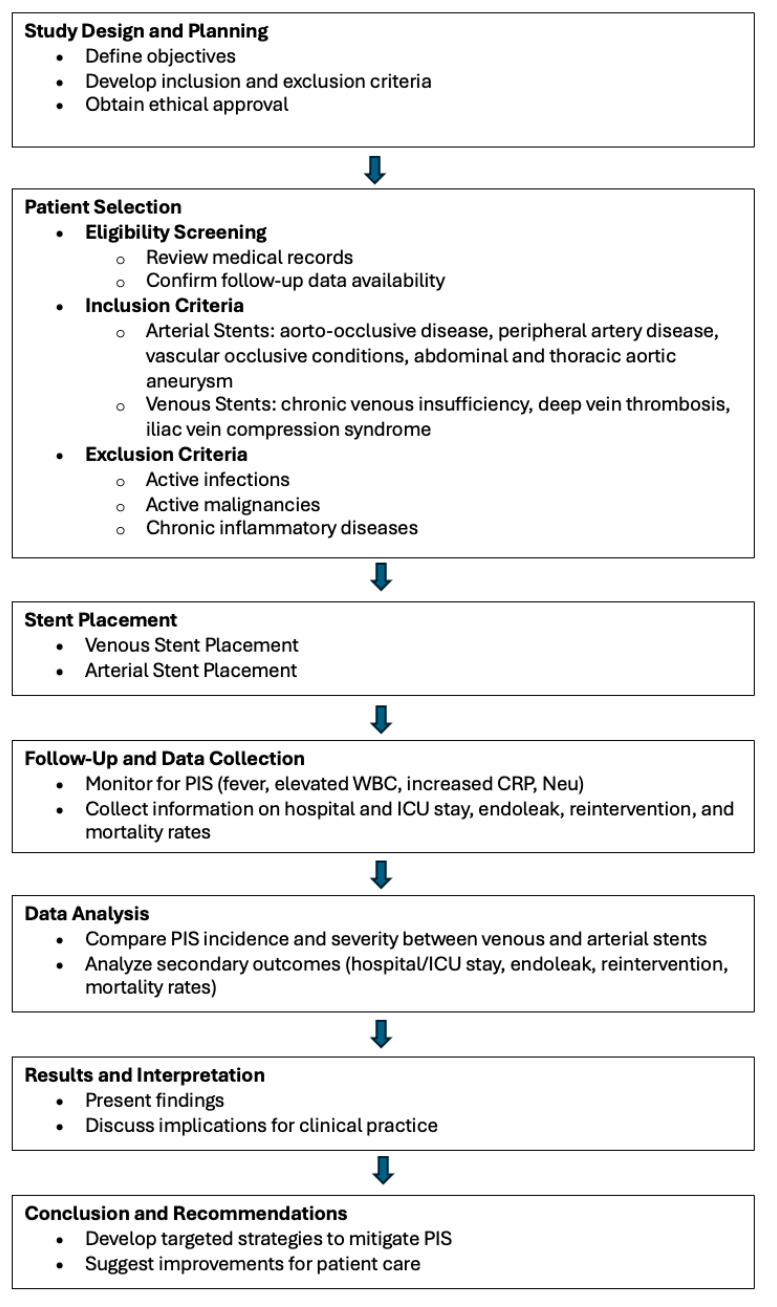
Study design and process.

Patients were selected based on the availability of complete medical records and follow-up data. Inclusion criteria for arterial stent placement included patients undergoing stenting for aorto-occlusive disease, peripheral artery disease, or other vascular occlusive conditions. For venous stenting, patients with indications such as chronic venous insufficiency, deep vein thrombosis, or iliac vein compression syndrome were included. Exclusion criteria for both groups involved patients with active infections, malignancies, or chronic inflammatory diseases that could confound the assessment of PIS (Figure 1).

The flowchart illustrates the study’s overall process from design to conclusion. It begins with the planning phase, where study objectives and criteria are defined, followed by patient selection based on inclusion and exclusion criteria. The flow proceeds through the stent placement phase, separating venous and arterial stents, and then into the follow-up and data collection stage. The data analysis compares outcomes between different stent types, leading to the interpretation of results. Finally, the chart concludes with recommendations for clinical practice based on the study’s findings.

### 2.2. Data Collection and Variables

Data were collected retrospectively from institutional electronic health records (EHRs). The following demographic and clinical variables were recorded: age, sex, comorbidities (e.g., diabetes mellitus, hypertension, hyperlipidemia), smoking status, and procedural details, including stent type, size, and location. For each patient, data regarding the development of PIS were collected, with PIS being defined by the presence of fever (>38 °C), elevated white blood cell (WBC) count (>12,000 cells/μL), or elevated C-reactive protein (CRP) levels (>10 mg/L) within 72 h post-procedure.

### 2.3. Post-Implantation Syndrome Assessment

The primary outcome measure was the incidence of PIS in both venous and arterial stent groups. The diagnosis of PIS was made based on clinical and laboratory findings. Fever was defined as an axillary temperature >38 °C measured on two consecutive occasions within 48–72 h post-procedure. Laboratory parameters, such as WBC count and CRP levels, were recorded pre-procedurally and at 24, 48, 72, 96, and 120 h post-stent placement. Any additional signs of systemic inflammation, including malaise, tachycardia, or hypotension, were also evaluated.

### 2.4. Stent Types and Material Properties

In the realm of vascular interventions, various stent brands are employed, each offering unique material properties that influence performance and clinical outcomes. PTFE and polyester are common materials used in stent grafts due to their durability and biocompatibility. In our study, we utilized several stent brands, including Endurant and Minerva, both manufactured by Medtronic in Ireland, which leverage PTFE to mitigate inflammatory responses. Additionally, Bentley stents, produced by Bentley InnoMed GmbH in Germany, utilize polyester for flexibility and strength in treating peripheral vascular diseases.

The Ankura stent (Shenzhen, China), developed by Lifetech, is specifically designed for thoracic aortic interventions, while the Myra stent (India), manufactured by Meril Life Sciences, is recognized for its hybrid architecture in various peripheral applications. The Castor stent, produced by Endovastec, is notable for its design aimed at treating thoracic aortic conditions, particularly aortic dissection involving arch lesions. It is the world’s first branched stent graft capable of simultaneously repairing the aorta and supra-arch branch arteries through minimally invasive techniques.

Lastly, the Atropos stent (Dublin, Ireland), also manufactured by Medtronic, is designed for various vascular conditions, enhancing flexibility and adaptability during deployment. The Abre stent (Dublin, Ireland), also from Medtronic, is a non-covered nitinol stent utilized in our study specifically for venous interventions. Unlike PTFE and polyester stents, the Abre stent’s design minimizes complications, such as migration and postimplantation syndrome (PIS). This differentiation in material usage underscores the importance of selecting appropriate stent types based on the specific clinical scenarios presented in vascular procedures [6,7,8].

### 2.5. Statistical Analysis

All continuous variables were assessed for normality with the Shapiro–Wilk test and by visual inspection of histograms. Variables following a normal distribution were expressed as means and standard deviations, while non-parametric variables were reported as medians with interquartile ranges. Differences between groups were tested using the Kruskall–Wallis test and the Mann–Whitney U test, as appropriate. Categorical variables were expressed as percentages and analyzed using the chi-squared test. A *p*-value of less than 0.05 was considered statistically significant. All tests were two-tailed, and statistical analyses were performed using SPSS software (Version 20.0, Chicago, IL, USA).

## 3. Results

A total of 191 patients were included in this analysis, with 36 patients receiving venous stents and 155 patients receiving arterial stents. Among those with arterial stents, 63 had PTFE grafts and 92 had polyester grafts. The mean age was comparable across the groups, with patients in the venous stent group having an average age of 51.25 ± 13.35 years, while those in the arterial stent group had an average age of 66.5 ± 11.7 years. When comparing PTFE and polyester grafts, there was no significant difference in age between the two groups (65.51 ± 10.81 vs. 67.25 ± 12.32 years, *p* = 0.503).

The proportion of male patients was significantly higher in the arterial stent group compared to the venous stent group (89.67% vs. 61.11%, *p* < 0.001). Among those with arterial stents, the proportion of males was slightly higher in the PTFE group (92.06%) compared to the polyester group (88.04%), although this difference was not statistically significant (*p* = 0.251).

The distribution of initial diagnoses varied significantly between venous and arterial stent patients (*p* < 0.001). All patients in the venous stent group were treated for deep vein thrombosis (DVT), whereas the arterial stent group had a mix of patients with peripheral arterial disease (PAD) (72.25%), abdominal aortic aneurysm (AAA) (23.22%), and thoracic aortic aneurysm (TAA) (4.51%). Among those with arterial stents, the PTFE group had a higher proportion of patients with PAD (84.13%) compared to the polyester group (64.13%). The prevalence of AAA was higher in the polyester group (29.35% vs. 14.29%, *p* = 0.005).

The prevalence of risk factors and comorbidities, including previous coronary artery disease (CAD), chronic obstructive pulmonary disease (COPD), hypertension (HTN), diabetes mellitus (DM), and malignancy, was similar across all groups. There were no statistically significant differences between PTFE and polyester grafts in terms of these comorbidities, including the prevalence of hypertension (26.98% vs. 39.13%, *p* = 0.330).

Preoperative white blood cell (WBC) counts were not significantly different between venous and arterial stent groups (*p* = 0.069) or between PTFE and polyester grafts (*p* = 0.715). Other preoperative parameters, including neutrophil counts, C-reactive protein (CRP) levels, platelet counts, and hematocrit (Hct) levels, also showed no significant differences between PTFE and polyester grafts. Postoperatively, both groups experienced similar trends in laboratory values over time, with no significant differences between PTFE and polyester stents in terms of WBC, neutrophil, CRP, platelet, or hematocrit levels at any time point.

Procedure times were longer in the venous stent group compared to the arterial stent group (35.1 ± 6.3 min vs. 30.1 ± 1.94 min, *p* = 0.018). However, there was no significant difference between PTFE and polyester grafts in arterial stents (33.24 ± 3.63 min vs. 27.99 ± 2.82 min, *p* = 0.251). The contrast medium volume used during the procedure was also similar between the two graft types (103.40 ± 6.91 mL for PTFE vs. 95.04 ± 5.81 mL for polyester, *p* = 0.310).

Hospital stay was significantly longer for patients with PTFE grafts compared to polyester grafts (6.15 ± 0.8 days vs. 4.51 ± 0.32 days, *p* < 0.001). Additionally, ICU stay duration was longer in the PTFE group (2.22 ± 0.7 days vs. 1.24 ± 0.15 days, *p* = 0.001).

There was no significant difference in the incidence of type I or type II endoleaks between PTFE and polyester grafts (4.76% vs. 3.26%, *p* = 0.374). Similarly, the 30-day reintervention rate was higher in the PTFE group (19.04% vs. 8.69%), but this difference did not reach statistical significance (*p* = 0.283). The 30-day mortality rate was higher in the polyester group (3.26% vs. 1.58%, *p* = 0.033), suggesting a potentially increased risk of early mortality with polyester grafts (Table 1).

This table compares demographic information and outcomes for patients receiving venous and arterial stents, as well as PTFE and polyester stents. The data include patient age, gender, comorbidities, and follow-up durations. Additionally, the table presents post-implantation syndrome (PIS) incidence, hospital and ICU stay durations, endoleak rates, reintervention rates, and mortality rates for each stent type. Values are presented as means (± standard deviation) for continuous variables and frequencies (percentages) for categorical variables.

In conclusion, patients with arterial stents experienced higher rates of PIS, as indicated by elevated WBC, neutrophil, and CRP levels, as well as longer hospital stays and higher postoperative complications such as endoleak. These findings suggest that arterial stent placement may be associated with a greater inflammatory response compared to venous stent placement.

In patients who developed PIS, our treatment approach primarily focused on managing the inflammatory response and associated symptoms. Antipyretics, such as paracetamol, were administered to control fever, while intravenous fluids were used to maintain adequate hydration and stabilize hemodynamic parameters. In cases of significant leukocytosis or elevated C-reactive protein (CRP) levels, a short course of broad-spectrum antibiotics was initiated to rule out any potential infectious component. Regular monitoring of vital signs, laboratory parameters, and clinical symptoms was carried out to assess the progression of PIS. Additionally, patients with prolonged or severe symptoms were evaluated for potential complications, such as endoleak or thrombus formation, which may have required further intervention or imaging studies.

## 4. Discussion

Postimplantation syndrome (PIS) is a systemic inflammatory response that occurs following the implantation of stents or endografts, typically characterized by fever, leukocytosis, and elevated inflammatory markers, such as C-reactive protein (CRP) in the absence of infection. In our study, we examined the incidence of PIS in patients undergoing venous and arterial stent placements, and our findings demonstrate significant differences in the inflammatory responses between these two groups. These results are consistent with the existing literature on PIS following endovascular aneurysm repair (EVAR), which highlights variations in PIS incidence based on procedural type, stent material, and patient characteristics [9].

Our analysis revealed that arterial stent patients were more prone to developing PIS, with higher postoperative WBC and neutrophil counts, along with more pronounced CRP elevation, when compared to patients with venous stents. This finding corroborates previous studies that suggest polyester grafts, often used in arterial stents, are associated with higher rates of PIS due to their proinflammatory properties. In contrast, venous stents, which typically utilize less inflammatory materials such as PTFE, exhibited a lower incidence of PIS, highlighting the influence of graft composition on postoperative outcomes [10]. 

### 4.1. Inflammatory Response and PIS Pathophysiology

The pathophysiology of PIS remains poorly understood, but the prevailing hypothesis is that the inflammatory response is triggered by the interaction between the stent material and the vascular endothelium, leading to the release of proinflammatory cytokines, such as interleukin-6 (IL-6) and tumor necrosis factor-alpha (TNF-α). In our study, arterial stent patients experienced more significant and sustained inflammatory responses, as evidenced by elevated CRP and neutrophil counts over several days postoperatively. This aligns with studies that have demonstrated a strong association between polyester grafts and heightened inflammatory reactions, as polyester is known to provoke a more robust foreign body response compared to PTFE grafts [11,12,13,14]. 

Moreover, our findings suggest that the inflammatory response following arterial stent placement is not only more severe but also more prolonged. Fever, a key marker of PIS, was more frequently observed in arterial stent patients, particularly at 72 and 96 h postoperatively. This prolonged febrile response is indicative of sustained systemic inflammation, which may be due to the larger surface area of the stent and the nature of the graft material, as well as the underlying vascular pathology being treated (e.g., arterial occlusive disease). These factors likely contribute to the increased incidence of PIS in arterial stent patients, as previously suggested by studies on EVAR [9]. 

### 4.2. Clinical Implications and Patient Outcomes

The clinical impact of PIS on patient outcomes has been the subject of debate. While some studies suggest that PIS is a transient and benign condition, our study demonstrates that PIS in arterial stent patients is associated with longer hospital stays and higher rates of postoperative complications, such as endoleaks and reintervention. Although PIS was not directly correlated with increased mortality or major adverse cardiac events (MACEs) in our cohort, its association with prolonged hospitalization and greater resource utilization underscores the clinical importance of early recognition and management of PIS [14,15]. 

Previous studies have suggested that the type of stent graft used may influence the long-term outcomes of patients with PIS. For instance, polyester grafts, which were more commonly used in our arterial stent group, have been associated with higher rates of inflammation and a greater risk of complications, such as endoleaks and stent-related infections. This is consistent with our findings, where patients with arterial stents experienced more frequent endoleaks and required more reinterventions compared to those with venous stents. The proinflammatory nature of polyester grafts may also explain the higher incidence of fever and leukocytosis in these patients [16,17].

### 4.3. Role of Graft Material and Procedure Type

The influence of graft material on the development of PIS is well-documented in the literature, with polyester being linked to a more pronounced inflammatory response than PTFE. Our study supports this, as arterial stent patients, many of whom received polyester grafts, had significantly higher levels of inflammatory markers and experienced more complications than venous stent patients, who were more likely to receive PTFE grafts [18].

These findings highlight the importance of considering graft material when planning endovascular interventions, as the choice of graft may have a significant impact on the postoperative inflammatory response and the risk of PIS [12,13,16].

Additionally, the duration and complexity of the procedure may also contribute to the development of PIS. Our data showed that arterial stent procedures, which tend to be more complex and longer in duration than venous stent procedures, were associated with a higher incidence of PIS. This is consistent with previous research suggesting that longer procedural times and the use of larger volumes of contrast media may increase the risk of PIS [4,19]. 

### 4.4. Role of Vascular Pathology in Post-Implantation Syndrome: Arteries vs. Veins

In addition to the material properties of the stents used, the underlying pathology of the vessels plays a significant role in the inflammatory response seen after endovascular interventions. Atherosclerosis, which is the primary disease process affecting arteries requiring stenting, is characterized by chronic inflammation. The inflammatory theory of atherosclerosis posits that the arterial wall becomes an active source of inflammatory mediators long before the intervention, contributing to a heightened inflammatory milieu even before stent placement [20]. This pre-existing inflammation likely exacerbates the post-implantation inflammatory response, thus contributing to a more pronounced presentation of PIS in arterial stenting. Conversely, thrombotic veins, though subject to clot formation, do not exhibit the same degree of chronic inflammation as the atherosclerotic arteries. While thrombus formation can trigger an acute inflammatory response, it is typically not a long-standing inflammatory process like atherosclerosis. As a result, venous stenting may lead to a less intense inflammatory reaction, and subsequently, a different PIS profile compared to arterial interventions. This fundamental difference between the inflammatory states of arteries and veins highlights the importance of considering vessel pathology when managing PIS, in addition to focusing on the stent material itself [21]. 

### 4.5. Role of Stent Migration in Post-Implantation Syndrome

Stent migration, a known complication, can exacerbate postimplantation syndrome (PIS) by triggering an inflammatory response due to vessel injury and mechanical irritation. The displacement of the stent can lead to further vascular damage, which may trigger or intensify the inflammatory cascade associated with PIS. In our study, no instances of stent migration were observed, largely due to the selection of stents longer than 10 cm. Shorter stents are more prone to migration due to insufficient anchorage within the vessel. By utilizing longer stents, we ensured better fixation, thereby reducing the risk of migration and its potential contribution to PIS. This strategic approach minimized both migration-related complications and the severity of PIS in our patient population [22,23]. 

### 4.6. Management of PIS and Future Research Directions

Given the association between PIS and adverse postoperative outcomes, effective management strategies are critical. In our study, patients with arterial stents required longer hospital stays, likely due to the need for close monitoring of inflammatory markers and management of complications such as endoleaks. Current management of PIS is largely supportive, focusing on hydration, antipyretics, and, in some cases, the use of corticosteroids or non-steroidal anti-inflammatory drugs (NSAIDs). However, the use of systemic anti-inflammatory medications remains controversial due to their potential side effects, particularly in high-risk patients [24,25,26]. 

Looking forward, targeted therapies that modulate the inflammatory response without broadly suppressing the immune system, such as interleukin inhibitors, may offer a promising approach to managing PIS. Further research is needed to identify specific biomarkers that can predict the development of PIS and guide treatment decisions. Additionally, the development of standardized diagnostic criteria for PIS would help clinicians better identify and manage this condition, reducing the risk of misdiagnosis and improving patient outcomes [27].

## 5. Limitations

Several limitations should be considered when interpreting our results. Variability in patient characteristics, including underlying comorbidities and concurrent medications, may influence the inflammatory response and complicate direct comparisons between venous and arterial stenting. A major limitation of this study is its retrospective design, which hinders the ability to assess the severity of inflammation in real-time. Prospective studies are recommended to provide more precise evaluations of the inflammatory response. The retrospective nature of some information collection may introduce biases. Future prospective studies with standardized protocols and controlled variables will help address these limitations and strengthen the evidence base.

## 6. Conclusions

In conclusion, our study highlights the significant differences in the incidence and severity of PIS between patients receiving venous and arterial stents. The findings suggest that arterial stent placement, particularly with polyester grafts, is associated with a more pronounced and prolonged inflammatory response, leading to increased postoperative complications and longer hospital stays. While PIS did not directly correlate with increased mortality or MACE in our study, its impact on patient recovery and resource utilization is substantial. 

This study demonstrated that arterial stent placement is associated with a higher incidence of postimplantation syndrome (PIS), characterized by elevated inflammatory markers, prolonged hospital stays, and an increased rate of postoperative complications, such as endoleaks and reinterventions. Polyester grafts, commonly used in arterial stenting, appear to provoke a stronger inflammatory response compared to PTFE grafts used in venous stents. However, although the incidence of PIS was lower in patients receiving venous stents, the presence of PIS should not be disregarded in these cases, as it still has the potential to affect recovery and clinical outcomes. Recognizing the risk of PIS in both venous and arterial stent placements is crucial for improving patient management. Early identification and appropriate management of PIS can help mitigate its impact, regardless of the type of stent used. This emphasizes the need for careful postoperative monitoring in all stent patients to optimize recovery and minimize complications.

Future research should focus on refining diagnostic criteria for PIS and exploring targeted therapies to mitigate the inflammatory response and improve patient outcomes.

## Figures and Tables

**Table 1:** Demographic and Clinical Outcome Insights: A Comparative Analysis of Venous vs. Arterial Stents and PTFE vs. Polyester Stents.

Variables	Venous Stents (n:36)	Arterial Stents (n:155)	*p* Value (Venous vs. Arterial)	Arterial Stents		*p* Value (PTFE vs. Polyester)
				PTFE Grafts (n:63)	POLYESTER Grafts (n:92)	
Demographical data						
Age (years)						
(mean ± SD)	51.25 ± 13.35	66.5 ± 11.7	0.463	65.51 ± 10.81	67.25 ± 12.32	0.503
Male						
(n, %)	22 (61.11%)	139(89.67%)	<0.001	58(92.06%)	81 (88.04%)	0.251
Initial diagnosis (n,%)			<0.001			0.005
PAD	0 (0.00%)	112(72.25%)		53(84.13%)	59 (64.13%)	
DVT	36 (100%)	0 (0.00%)		0 (0.00%)	0 (0.00%)	
AAA	0 (0.00%)	36 (23.22%)		9 (14.29%)	27 (29.35%)	
TAA	0 (0.00%)	7 (4.51%)		1 (1.59%)	6 (6.52%)	
Risk factors/ Comorbidities (n,%)			0.077			0.330
Previous CAD	2 (5.55%)	22 (14.19%)		0 (0.00%)	1 (1.09%)	
PreviousCOPD	2 (5.55%)	13 (8.38%)		1 (1.59%)	1 (1.09%)	
Previous HTN	4(11.11%)	49 (31.61%)		17 (26.98%)	36 (39.13%)	
Previous DM	4(11.11%)	45 (29.03%)		23 (36.50%)	26 (28.26%)	
Previous malignancy	1 (2.77%)	6 (3.87%)		2 (3.17%)	5 (5.43%)	
Preoperative laboratory results						
WBC (×10^9^/L) (mean ± SD)	6.75 ± 2.02	8.84 ± 3.13	0.069	8.97 ± 3.02	8.75 ± 3.23	0.715
Neu (×10^9^/L) (mean ± SD)	4.54 ± 2.04	6.18 ± 3.14	0.060	6.38 ± 3.09	6.04 ± 3.11	0.847
CRP (mg/L) (mean ± SD)	12.24 ± 2.75	21.48 ± 3.55	0.052	21.05 ± 3.12	21.78 ± 4.63	0.964
PLT (×10^9^/L) (mean ± SD)	253 ± 19.01	260 ± 8.42	0.948	254.16 ± 12.21	264.96 ± 8.4	0.974
Hct (%) (mean ± SD)	37.5 ± 7.16	39.2 ± 7.56	0.443	39.14 ± 8.15	39.26 ± 7.18	0.531
Procedure Time (min) (mean ± SD)	35.1 ± 6.3	30.1 ± 1.94	0.018	33.24 ± 3.63	27.99 ± 2.82	0.251
Contrast medium volume (mL) (mean ± SD)	86.7 ± 8.5	98.4 ± 4.32	0.251	103.40 ± 6.91	95.04 ± 5.81	0.310
Postoperative laboratory results						
WBC (×10^9^/L) (mean ± SD)						
24 h	7.21 ± 2.75	10.16 ± 4.48	0.046	10.09 ± 4.10	10.21 ± 4.74	0.642
48 h	7.08 ± 2.62	9.96 ± 3.57	0.014	9.68 ± 3.79	10.15 ± 3.41	0.520
72 h	7.46 ± 3.02	9.82 ± 3.17	0.539	9.73 ± 3.12	9.88 ± 3.22	0.893
96 h	6.98 ± 2.61	9.12 ± 2.93	0.704	8.87 ± 3.00	9.27 ± 2.84	0.630
120 h	6.62 ± 2.49	8.82 ± 2.96	0.721	8.75 ± 3.02	8.87 ± 2.93	0.985
Neu (×10^9^/L)						
(mean ± SD)						
24 h	5.05 ± 2.07	7.76 ± 4.12	<0.001	7.83 ± 4.29	7.71 ± 3.98	0.350
48 h	4.72 ± 1.92	7.49 ± 3.93	0.004	7.36 ± 3.70	7.58 ± 4.09	0.769
72 h	5.16 ± 2.39	7.06 ± 3.14	0.514	6.97 ± 2.98	7.12 ± 3.26	0.945
96 h	4.50 ± 1.96	6.76 ± 2.97	0.067	6.51 ± 2.77	6.93 ± 3.10	0.465
120 h	4.83 ± 1.82	6.37 ± 2.96	0.154	6.28 ± 3.17	6.43 ± 2.82	0.506
CRP (mg/L)						
(mean ± SD)						
24 h	36.34 ± 6.21	41.41 ± 4.01	0.203	36.43 ± 4.74	44.83 ± 4.85	0.324
48 h	50.91 ± 8.62	74.72 ± 5.32	0.007	68.00 ± 7.24	79.39 ± 6.17	0.486
72 h	44.72 ± 7.22	77.93 ± 5.31	0.004	76.70 ± 7.89	78.71 ± 8.03	0.743
96 h	36.81 ± 6.24	66.51 ± 4.94	0.004	69.15 ± 7.41	64.77 ± 6.32	0.222
120 h	21.00 ± 4.53	46.93 ± 4.73	<0.001	47.4 ± 6.43	46.6 ± 8.02	0.774
PLT (×10^9^/L)						
(mean ± SD)						
24 h	232.6 ± 17.31	243.4 ± 7.52	0.719	244.72 ± 11.52	242.62 ± 8.57	0.568
48 h	215.8 ± 13.92	235 ± 7.41	0.457	245.59 ± 11.02	227.85 ± 8.24	0.508
72 h	226.2 ± 13.91	232.3 ± 6.94	0.816	238.54 ± 11.34	228.03 ± 7.62	0.245
96 h	234.2 ± 14.13	246.2 ± 6.72	0.799	253.06± 9.32	241.61± 8.06	0.529
120 h	236 ± 84.94	261.3 ± 6.91	0.753	273.06 ± 10.71	253.63 ± 7.51	0.086
Hct (%)						
(mean ± SD)						
24 h	35.7 ± 7.09	36.3 ± 7.04	0.260	32.24 ± 7.50	36.45 ± 6.76	0.358
48 h	35.8 ± 6.42	34.8 ± 7.35	0.148	34.53 ± 6.99	35.04 ± 7.62	0.364
72 h	36.0 ± 6.17	34.3 ± 7.05	0.228	33.70 ± 6.74	34.76 ± 7.26	0.374
96 h	37.1 ± 4.49	34.3 ± 7.05	0.026	34.34 ± 6.50	34.29 ± 7.44	0.869
120 h	36.4 ± 5.46	35.7 ± 6.42	0.399	35.57 ± 6.75	35.87 ± 6.18	0.175
Postoperative Body Temperature (°C)						
(mean ± SD)						
24 h	37.2 ± 0.34	37.4 ± 0.37	0.547	37.43 ± 0.36	37.41 ± 0.37	0.526
48 h	37.4 ± 0.77	37.7 ± 0.78	0.211	37.75 ± 0.76	37.61 ± 0.81	0.385
72 h	37.1 ± 0.45	37.2 ± 0.41	0.437	37.24 ± 0.40	37.12 ± 0.43	0.430
96 h	37.0 ± 0.01	37.0 ± 0.01	0.997	37.24 ± 0.40	37.21 ± 0.42	0.697
120 h	36.5 ± 0.05	36.5 ± 0.04	0.840	37.00 ± 0.00	37.00 ± 0.00	0.896
Hospital stay, days	3.42 ± 0.36	5.59 ± 0.46	0.018	6.15 ± 0.8	4.51 ± 0.32	<0.001
ICU stay, days	1.03 ± 0.16	1.78 ± 0.38	0.061	2.22 ± 0.7	1.24 ± 0.15	0.001
30-day endoleak (n, %) Type I Type II			*			0.374
			
	0 (0.00%)	1 (0.64%)		0 (0.00%)	1 (1.09%)	
	0 (0.00%)	7 (4.51%)		3 (4.76%)	3 (3.26%)	
30-day reintervention (n, %)	4 (1.1%)	16 (10.32%)	0.783	12 (19.04%)	8 (8.69%)	0.283
30-day mortality (n, %)	0 (0%)	4 (2.58%)	0.071	1 (1.58%)	3 (3.26%)	0.033

Abbreviations: PAD: peripheral arterial disease, DVT: deep vein thrombosis, AAA: abdominal aortic aneurysm, TAA: thoracic aortic aneurysm, CAD: coronary artery disease, COPD: chronic obstructive pulmonary disease, HTN: hypertension, DM: diabetes mellitus, WBC: white blood cell. CRP: C-reactive protein, Hct: hematocrit, PLT: platelet count. h: hour, ICU: intensive care unit). *: No *p*-value is reported for venous stents due to the absence of endoleak cases. A *p*-value less than 0.05 is generally considered statistically significant.

## Data Availability

The data presented in this study are available on request from the corresponding author due to privacy and ethical reasons.
